# Neuroimaging in Tick Paralysis: Looking Outside the Box

**DOI:** 10.3390/idr14060085

**Published:** 2022-11-11

**Authors:** Zereen Sarwar, Aaron Fletcher Osborne, Chetan Shah, Mobeen H. Rathore

**Affiliations:** 1 Department of Radiology, College of Medicine, University of Florida, Gainesville, FL 32601, USA; 2 Department of Pediatrics, Center for HIV/AIDS Research, Education and Service (UF CARES), University of Florida, Jacksonville, FL 32209, USA; 3 Department of Pediatric Radiology, Nemours Children’s Health, Jacksonville, FL 32207, USA

**Keywords:** tick paralysis, ticks, paralysis, *Dermacentor variabilis*, imaging, MRI

## Abstract

Tick paralysis is a rare but potentially deadly form of muscle paralysis caused by a neurotoxin transmitted through the saliva of gravid, engorged female ticks of various species. Often, there is an initial misdiagnosis or delay in diagnosis due to the rarity of the diagnosis, the obscure location of the tick, and the disease’s clinical similarity to Guillain–Barre syndrome. We report the case of a 4-year-old girl with tick paralysis in whom the tick was not found until 2 days after hospital admission. Upon the review of the imaging, it was discovered that the tick was visible on the MRI of the brain that had been reported as normal.

## 1. Introduction

Tick paralysis (TP) is an uncommon disease caused by a neurotoxin produced by the gravid, engorged females of certain tick species [1,2,3]. It most often occurs in North America and Australia during the spring and summer seasons, though sporadic cases have been reported in Europe and Africa as well [1,2]. Over 70 species of ticks have been identified that can cause TP (3). *Dermacentor andersoni*, the Rocky Mountain wood tick, predominates in the northwestern and western US, as well as in southwestern Canada [1]. In the southeastern US, the usual culprit is *Dermacentor variabilis*, the American dog tick [1]. In Australia, *Ixodes holocyclus*, the marsupial tick, also referred to as the “Australian paralysis tick”, is responsible for most cases [1,4]. 

The initial presentation of TP often consists of nonspecific flu-like symptoms such as malaise and generalized weakness. If the tick remains attached, progressive muscle weakness can occur, most often in an ascending pattern, and this can eventually lead to respiratory failure [1,2]. The prompt removal of the tick usually leads to improvements in the symptoms within a few hours and the resolution of the symptoms within days [1,2]. However, due to the rarity of the diagnosis, the location of tick bites in inconspicuous areas, and common misdiagnoses such as Guillain–Barre syndrome, tick removal is often delayed, leading to a worsening of the disease and potential death [1,2]. 

## 2. Case Presentation

A 4-year-old girl presented complaining of diffuse weakness of a one-day duration. The day before her presentation, the patient’s mother noticed that the patient was having difficulty ambulating. The mother subsequently took the patient to an outside emergency department. The CT scan of the head was normal, and the urine toxicology was negative. The patient was discharged home with a diagnosis of hip strain. 

The next morning, the patient was unable to stand and had significant weakness of all four extremities, which prompted the mother to bring the patient to our emergency department. She was in her normal state of health prior to this, and she did not have any other symptoms. 

The neurologic examination indicated the patient’s significantly decreased truncal tone, combined with the inability to hold her head up, sit up, or bear weight. She was able to move all four extremities, withdrew from pain, and had no facial asymmetry. Her patellar reflexes were faint on the right and unable to be elicited on the left. The remainder of the physical examination was within the normal limits. 

The MRI of the brain and cervical, thoracic, and lumbar spine through contrast enhancement were reported as normal.

The laboratory examinations, including a cerebrospinal fluid examination, were normal. 

**Figure 1:**
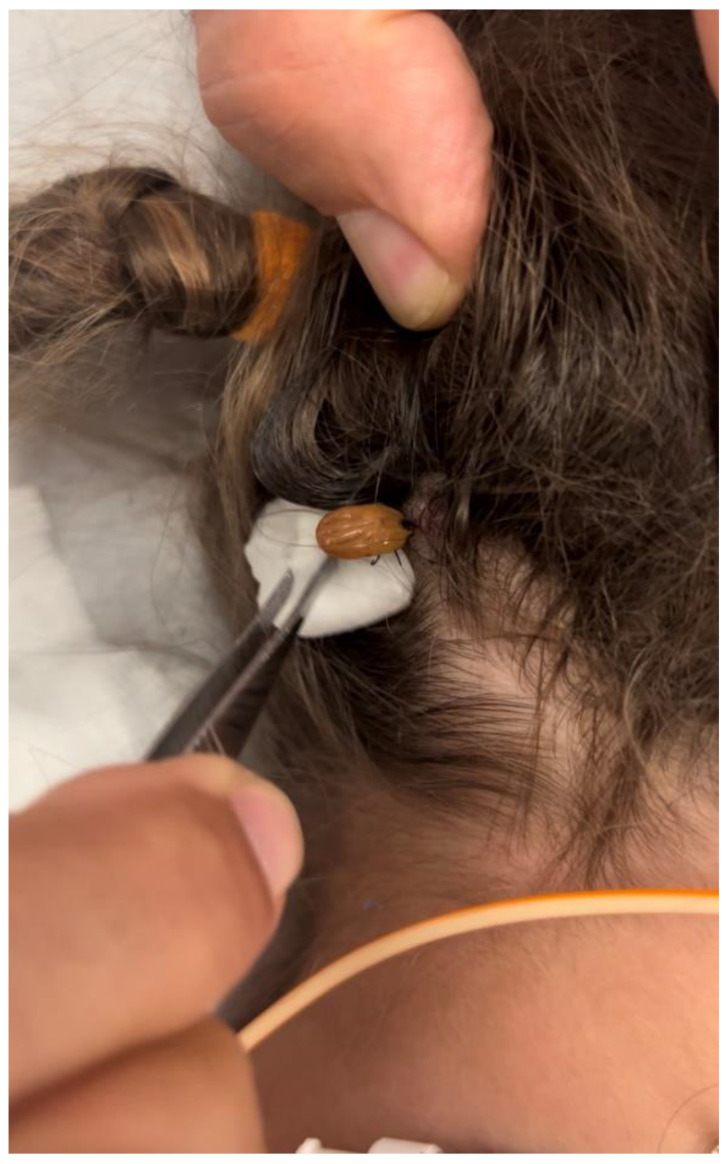
Engorged tick on the hairline.

The patient’s condition deteriorated over the next 2 days, with increasing weakness and difficulty in respiration requiring intubation. Plasmapheresis was also initiated. Two days after admission, a large, engorged tick was found by the mother (Figure 1) on the posterior aspect of the patient’s scalp under a clump of deeply matted hair. It was promptly removed, and doxycycline was initiated. Her condition quickly improved after the removal. She was extubated and started on BiPap within two days of the tick’s removal. The patient was discharged from the hospital 7 days after the tick’s removal.

The tickborne illness PCR panels for Anaplasma, Babesia, Borrelia, Ehrlichia, and Lyme disease were negative. The tick was identified as the American dog tick (*Dermacentor variabilis*).

**Figure 2:**
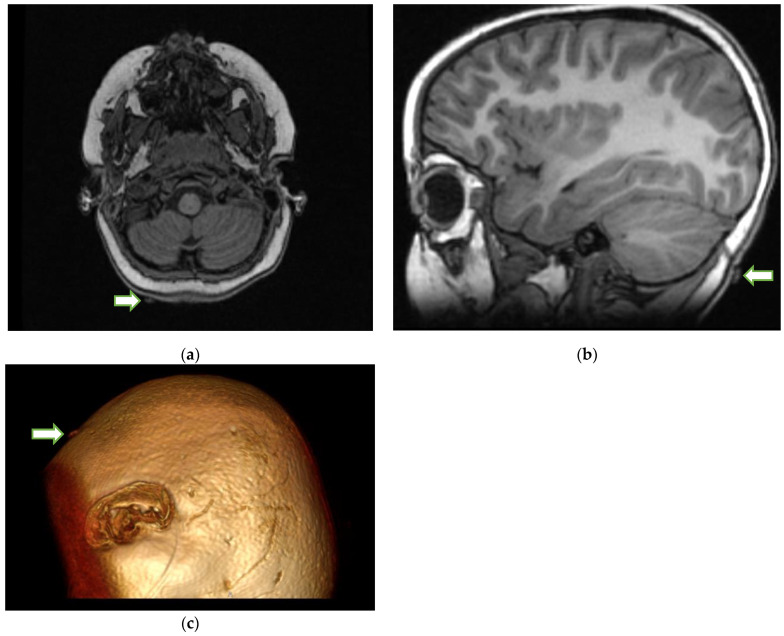
Axial T1-weighted (**a**) MRI of the brain at the level of the medulla shows a normal appearance of the medulla and the cerebellum. However, on closer inspection, there is a small, isointense area (arrow) seen attached to the skin in the occipital region on the right side. The arrow on the sagittal T1-weighted MRI of the brain (**b**) also points to the tick. The 3D rendering of the MRI (**c**) shows what appears to be a tick (arrow) on the surface of the head.

Upon review of the patient’s head imaging, it was determined that the tick could be seen on the MRI (Figure 2) of the brain performed earlier. 

## 3. Discussion

While TP is a rare diagnosis, the delayed recognition of the disorder can lead to dire consequences. There period of tick attachment required for blood meal and the secretion of paralytic neurotoxin is unknown (presumed to be 4–7 days, with a mean of 5 days). This neurotoxin acts by causing the presynaptic inhibition of acetylcholine release and eliminates the dependence of acetylcholine release on the levels of extracellular calcium. After this, the patient develops non-specific, influenza-like symptoms of malaise and weakness. This is followed by the onset of paralysis 1–2 days later (range = 1–10 days) [1,3,5]. When diagnosed and treated by prompt tick removal, the time to full neurological recovery is usually short, with a mean of 1.5 days (range = 1–2.5 days). Even after the tick’s removal, paralysis may last for a short time, and mechanical ventilation may still be required, as in the case of our patient.

There is one systematic review of 33 human cases identified between 1946 and 1966 and another meta-analysis encompassing a 60-year period in the US, reported in 2010 [2,6]. Overall, TP is more common in females (80% of cases), especially those under 8 years of age (68% of cases) [2,6]. This is thought to be due to the fact that females, on average, have longer hair, which can better conceal any ticks. The most frequent attachment site for ticks is on the head and scalp (48% of cases), particularly on the posterior neck hairline [2,6].

The search for a tick on the scalp should be performed on a patient with paralysis. An MRI or CT scan is usually performed for patients with weakness or paralysis. Typically, MRI and CT scans of the head are normal in the context of TP. A normal MRI of the brain in a patient with paralysis should prompt a search, especially in the occipital soft tissues, to identify a tick-shaped extraneous structure on the axial images of the brain. The search for a tick on the scalp can be a daunting task. Locating it on MR or CT may act as a guide to narrow the search area. Sometimes, the answer lies in the most minor of observations. Because the removal of the tick is the main treatment for TP, MRI identification and localization, especially with 3D reconstruction, may play a critical role in these patients, as the tick can be removed before the progressive paralysis causes respiratory failure or even death in rare cases. As TP is frequently misdiagnosed as GBS and other acute ascending motor neuropathies, early tick identification may also prevent further unnecessary diagnostic tests or treatments [1,2,7,8].

Once the tick is localized on MRI, the search of the patient’s hair becomes easier. Ticks should be removed carefully following the recommended technique, with clean, fine-tipped tweezers which are used to grasp the tick as close to skin as possible and then pull it upwards with a steady, even pressure, without twisting or jerking motions [9]. Care should be taken to ensure that all the mouthparts have been removed, as these contain salivary glands that may continue to secrete toxins even after the body of the tick has been removed [10]. 

## Data Availability

Not applicable.
